# Homocysteine, B vitamins, and cardiovascular disease: a Mendelian randomization study

**DOI:** 10.1186/s12916-021-01977-8

**Published:** 2021-04-23

**Authors:** Shuai Yuan, Amy M. Mason, Paul Carter, Stephen Burgess, Susanna C. Larsson

**Affiliations:** 1 Unit of Cardiovascular and Nutritional Epidemiology, Institute of Environmental Medicine, Karolinska Institutet, Nobelsväg 13, 17177 Stockholm, Sweden; 2 British Heart Foundation Cardiovascular Epidemiology Unit, Department of Public Health and Primary Care, University of Cambridge, Cambridge, UK; 3 National Institute for Health Research Cambridge Biomedical Research Centre, University of Cambridge and Cambridge University Hospitals, Cambridge, UK; 4 Department of Medicine, University of Cambridge, Cambridge, UK; 5 MRC Biostatistics Unit, University of Cambridge, Cambridge, UK; 6 Department of Public Health and Primary Care, University of Cambridge, Cambridge, UK; 7 Unit of Medical Epidemiology, Department of Surgical Sciences, Uppsala University, Uppsala, Sweden

**Keywords:** Cardiovascular disease, Homocysteine, Mendelian randomization, B vitamins

## Abstract

**Background:**

Whether a modestly elevated homocysteine level is causally associated with an increased risk of cardiovascular disease remains unestablished. We conducted a Mendelian randomization study to assess the associations of circulating total homocysteine (tHcy) and B vitamin levels with cardiovascular diseases in the general population.

**Methods:**

Independent single nucleotide polymorphisms associated with tHcy (*n* = 14), folate (*n* = 2), vitamin B6 (*n* = 1), and vitamin B12 (*n* = 14) at the genome-wide significance level were selected as instrumental variables. Summary-level data for 12 cardiovascular endpoints were obtained from genetic consortia, the UK Biobank study, and the FinnGen consortium.

**Results:**

Higher genetically predicted circulating tHcy levels were associated with an increased risk of stroke. For each one standard deviation (SD) increase in genetically predicted tHcy levels, the odds ratio (OR) was 1.11 (95% confidence interval (CI), 1.03, 1.21; *p* = 0.008) for any stroke, 1.26 (95% CI, 1.05, 1.51; *p* = 0.013) for subarachnoid hemorrhage, and 1.11 (95% CI, 1.03, 1.21; *p* = 0.011) for ischemic stroke. Higher genetically predicted folate levels were associated with decreased risk of coronary artery disease (OR_SD_, 0.88; 95% CI, 0.78, 1.00, *p* = 0.049) and any stroke (OR_SD_, 0.86; 95% CI, 0.76, 0.97, *p* = 0.012). Genetically predicted increased vitamin B6 levels were associated with a reduced risk of ischemic stroke (OR_SD_, 0.88; 95% CI, 0.81, 0.97, *p* = 0.009). None of these associations persisted after multiple testing correction. There was no association between genetically predicted vitamin B12 and cardiovascular disease.

**Conclusions:**

This study reveals suggestive evidence that B vitamin therapy and lowering of tHcy may reduce the risk of stroke, particularly subarachnoid hemorrhage and ischemic stroke.

**Supplementary Information:**

The online version contains supplementary material available at 10.1186/s12916-021-01977-8.

## Background

**Fig. 1:**
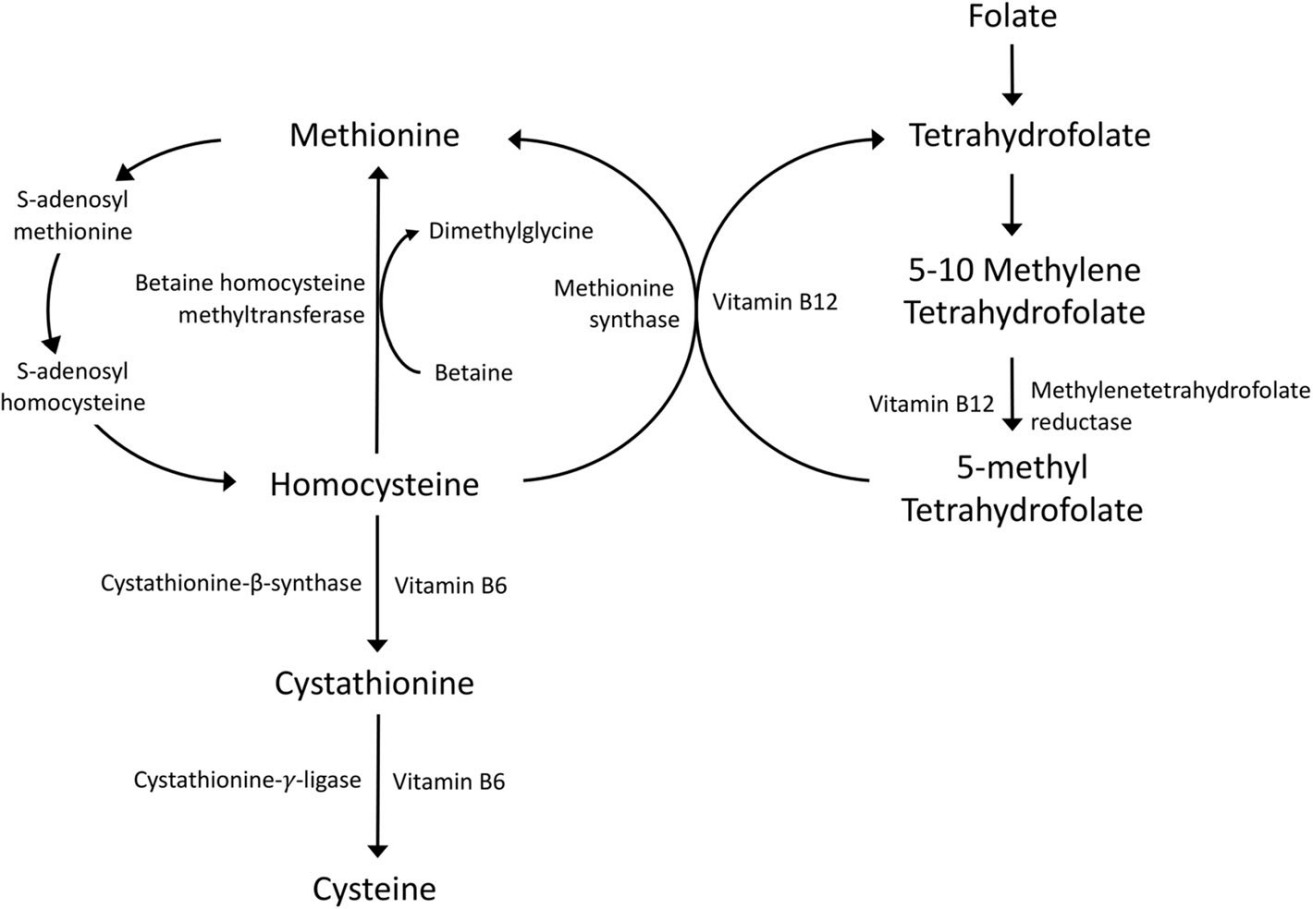
Overview of folate, vitamin B6, and vitamin B12 in homocysteine metabolism. Homocysteine is reconverted to methionine by receiving a methyl group from 5-methyltetrahydrofolate, the active form of folate, or betaine in the remethylation pathway. Irreversible removal of homocysteine occurs through the transsulphuration pathway where homocysteine condenses with serine to form cystathionine

The B vitamins, including folate and vitamins B6 and B12, play vital roles in the metabolism of homocysteine (Fig. 1) [1]. Deficiency of either of these B vitamins can lead to an elevated circulating level of total homocysteine (tHcy), which has been implicated in the development of cardiovascular disease (CVD) [2–5]. The association has been supported by several possible underlying pathophysiologic mechanisms, such as impaired endothelial function, increased oxidative stress, induced vascular inflammation, stimulated vascular smooth muscle cell proliferation, and activated coagulation factors by homocysteinemia [1]. However, randomized controlled trials (RCTs) have generally not detected a protective effect of homocysteine-lowering therapy with B vitamins on total CVD [6–8] or coronary artery disease [4, 6]. Findings of corresponding RCTs on stroke are inconclusive [9–12]. Potential explanations for the inconsistent results may be related to small sample sizes, low adherence to the treatment, and different study populations and CVD outcomes.

Using genetic variants as instrumental variables for an exposure (e.g., tHcy), the Mendelian randomization (MR) design can strengthen the causal inference by minimizing residual confounding and reverse causation. Previous MR studies showed that tHcy levels proxied by a single nucleotide polymorphism (SNP) in the *MTHFR* gene region were associated with stroke [13] and imaging burden of cerebral small vessel disease [14], but not with coronary artery disease [15, 16]. Studies utilizing more SNPs suggested a positive association of genetically predicted tHcy levels with risk of ischemic stroke, especially small vessel stroke [17], but no association with coronary artery disease [17–19] and atrial fibrillation [20]. Data are scarce for other cardiovascular diseases (CVDs).

Here, we conducted an MR study to assess the associations of genetically predicted tHcy levels with a wide range of CVDs. We also examined the associations of genetically predicted levels of folate and vitamins B6 and B12 with CVDs.

## Methods

### Outcome data sources

Summary-level data for 12 CVD endpoints were obtained from large genetic consortia [21–26], the UK Biobank study [27] and the FinnGen consortium [28]. Detailed descriptions on data sources are presented in Table 1.

**Table 1:** Information on outcome data sources

Data source	Cardiovascular disease	Population	Cases	Controls	Covariates adjusted in GWAS
Consortium (Nielsen et al.)	Atrial fibrillation	European	60,620	970,216	Birth year, sex, genotype batch, and one to four principal components
CARDIoGRAMplusC4D+UKBB	Coronary artery disease	Mixed	122,733	424,528	Not reported
HERMES consortium	Heart failure	European	47,309	930,014	Age and sex, and principal components in individual studies where applicable
MEGASTROKE consortium	Stroke		40,585	406,111	Age and sex
	Ischemic stroke		34,217	NA	
ISGC	Intracerebral hemorrhage	European	3223	3725	Age, sex, and principal components
Consortium (Bakker et al.)	Subarachnoid hemorrhage	European	7495	71,934	Not reported
The UK Biobank study (UKBB)	Aortic aneurysm	European	2261	365,300	Age, sex, and ten genetic principal components
	Aortic valve stenosis		3528	364,033	
	Stroke		12,036	355,525	
	Intracerebral hemorrhage		1504	366,057	
	Ischemic stroke		6566	360,995	
	Transient ischemic attack		4813	362,748	
	Venous thromboembolism		16,412	351,149	
	Peripheral vessel disease		4593	362,968	
The FinnGen consortium	Aortic aneurysm	European	1919	167,843	Age, sex, the first ten genetic principal components, and genotyping batch
	Atrial fibrillation		17,325	97,214	
	Coronary artery disease		16,631	160,268	
	Heart failure		9576	159,286	
	Stroke		14,171	133,027	
	Intracerebral hemorrhage		1224	163,533	
	Subarachnoid hemorrhage		1019	163,508	
	Ischemic stroke		8046	164,286	
	Transient ischemic attack		6729	164,286	
	Venous thromboembolism		6913	169,986	
	Peripheral vessel disease		5323	167,843	

*CARDIoGRAMplusC4D* Coronary ARtery DIsease Genome wide Replication and Meta-analysis plus The Coronary Artery Disease Genetics, *GWAS* genome-wide association study, *HERMES* Heart Failure Molecular Epidemiology for Therapeutic Targets, *ISGC* International Stroke Genetic Consortium, *NA* not available. The UK Biobank was included in Consortium (Nielsen et al.), HERMES consortium, ISGC, and Consortium (Bakker et al.)

### Instrument selection

SNPs associated with tHcy and B vitamins were identified at the genome-wide significance threshold (*p* < 5 × 10^−8^) from meta-analyses of genome-wide association studies on tHcy (*n* = 44,147 individuals) [29], folate (*n* = 37,465 individuals) [30], vitamin B6 (*n* = 1864 individuals) [31], and vitamin B12 (*n* = 45,576 individuals) [30] in individuals of European ancestry. Linkage disequilibrium among SNPs for one exposure was estimated using PLINK clumping method based on 1000 Genomes European reference panel. Independent SNPs without linkage disequilibrium (*r*^*2*^ *<* 0.01 and clump window > 10 kb) were used as instrumental variables (Supplementary Table 1). The SNPs explained 6.0% of variance for tHcy [29], 1.0% of variance for folate [30], 1.3% of variance for vitamin B6 [31], and 6.0% of variance for vitamin B12 [30]. Proxy SNPs (*r*^*2*^ > 0.8) were used for specific tHcy- or B vitamin-associated SNPs that were unavailable in outcome datasets. Missing SNPs without suitable proxies were excluded from analyses.

### Statistical analysis

We used the multiplicative random-effects inverse-variance-weighted model [32] as the main analysis. Estimates for one CVD endpoint from different sources were combined using the fixed-effects meta-analysis method. Three sensitivity analyses, including the weighted median [33], MR-Egger [34], and MR-PRESSO [35] approaches, were performed for tHcy and vitamin B12. The weighted median model generates consistent causal estimates assuming that more than a half of the weights derive from valid SNPs [33]. The MR-Egger regression can detect horizontal pleiotropy by *p* value for its intercept and provide estimate after correction for pleiotropic effects under the instrument strength independent of direct effect assumption although it consumes statistical power [34]. The MR-PRESSO method can detect outlying SNPs and provide causal estimates after removal of possible outliers under the assumption that the used SNPs are valid [35]. By searching phenotypes associated with used SNPs for tHcy in PhenoScanner V2 [36] (Supplementary Table 2), 4 SNPs (rs1047891, rs548987, rs2251468, and rs838133 [37]) associated with blood lipids and other traits were likely to exert pleiotropic effects. We performed additional sensitivity analysis with the exclusion of these 4 SNPs. The *I*^*2*^ statistic was calculated to assess the degree of heterogeneity [38] among estimates of SNPs in each analysis. Power was estimated using an online tool (Supplementary Table 3) [39]. The odds ratios (ORs) and corresponding 95% confidence intervals (CIs) of CVDs were scaled to one-standard deviation (SD) increase in genetically predicted circulating levels of tHcy and B vitamins. We used a conservative Bonferroni-based *p* value threshold of 0.001, accounting for 4 exposures and 12 outcomes. Associations with a *p* value between the Bonferroni-corrected significance level and the conventional significance level (i.e., < 0.05) were deemed as suggestive associations. All *p* values were two-sided and analyses were performed using the mrrobust package [40] in Stata/SE 15.0 and the TwoSampleMR package [41] in R Software 3.6.0.

## Results

**Fig. 2:**
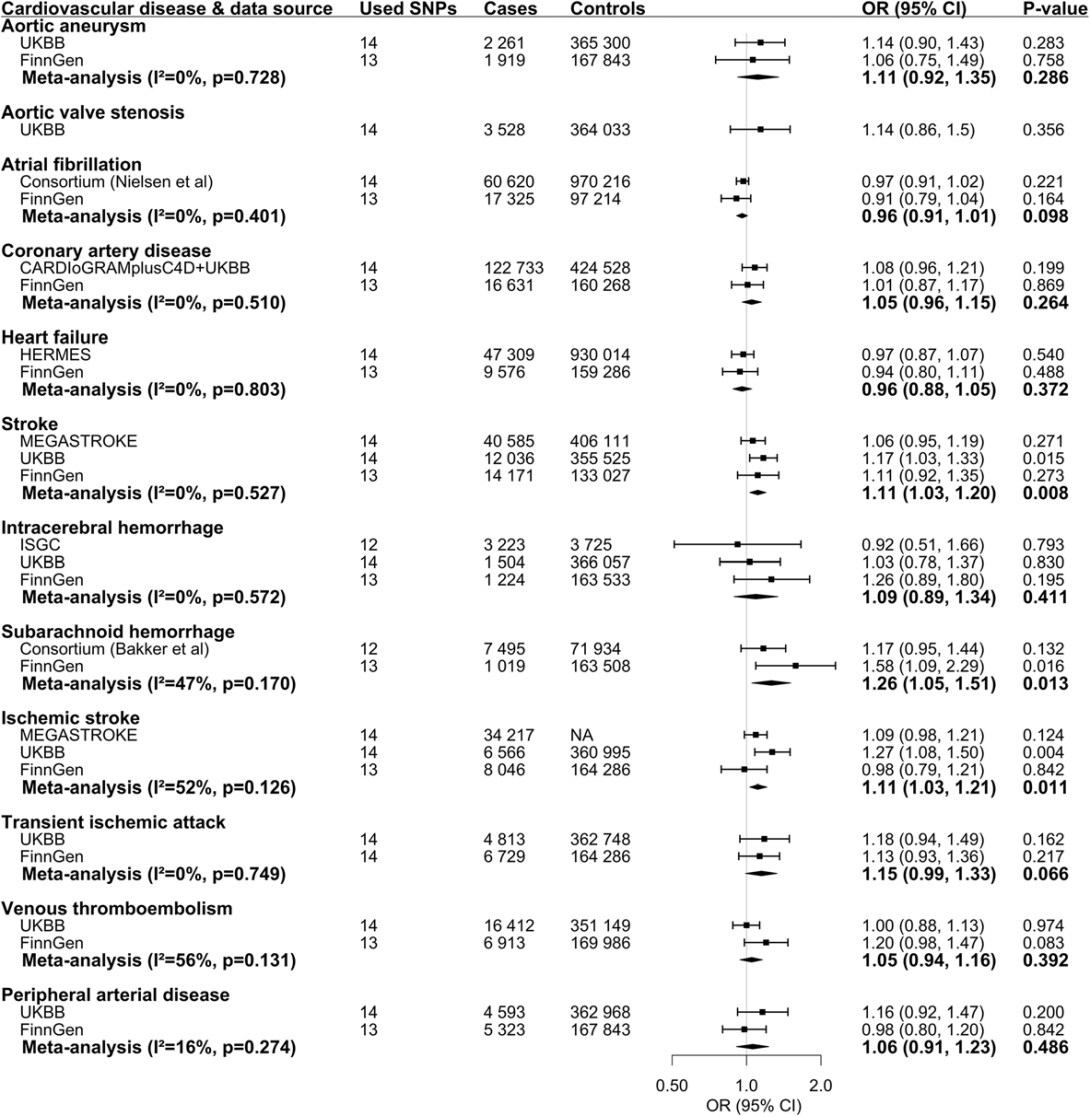
Associations of genetically predicted circulating homocysteine levels with risk of cardiovascular diseases. CARDIoGRAMplusC4D, Coronary ARtery DIsease Genome wide Replication and Meta-analysis plus The Coronary Artery Disease Genetics; CI, confidence interval; CVD, cardiovascular disease; HERMES; Heart Failure Molecular Epidemiology for Therapeutic Targets; ISGC, International Stroke Genetic Consortium; OR, odds ratio; UKBB, UK Biobank. The UK Biobank was included in Consortium (Nielsen et al.), HERMES consortium, ISGC, and Consortium (Bakker et al.)

We observed suggestive associations of higher genetically predicted circulating tHcy levels with increased risk of any stroke, subarachnoid hemorrhage, and ischemic stroke (Fig. 2). For 1-SD increase in genetically predicted tHcy levels, the combined OR was 1.11 (95% CI, 1.03, 1.21; *p* = 0.008) for stroke, 1.26 (95% CI, 1.05, 1.51; *p* = 0.013) for subarachnoid hemorrhage, and 1.11 (95% CI, 1.03, 1.21; *p* = 0.011) for ischemic stroke. Results remained directionally consistent in the weighted median model (Supplementary Table 4). We noticed moderate heterogeneity in the analyses of stroke and ischemic stroke and possible pleiotropy in the MR-Egger analysis for ischemic stroke in FinnGen (Supplementary Table 4). Genetically predicted tHcy levels were not associated with any of other studied CVDs (Fig. 2). One to three outliers were identified in the MR-PRESSO analysis. The results remained overall consistent in the MR-PRESSO analysis after the removal of outliers (Supplementary Table 5). In the sensitivity analysis with exclusion of 4 pleiotropic SNPs, the associations remained (Supplementary Table 6). For 1-SD increase in genetically predicted tHcy levels, the combined OR was 1.14 (95% CI, 1.04, 1.24; *p* = 0.003) for stroke, 1.26 (95% CI, 1.03, 1.53; *p* = 0.024) for subarachnoid hemorrhage and 1.15 (95% CI, 1.04, 1.27; *p* = 0.002) for ischemic stroke.

The associations of genetically predicted circulating B vitamins with CVDs are shown in Supplementary Figure 1 to 3. There were suggestive associations of higher genetically predicted folate levels with decreased risk of coronary artery disease (OR_SD_, 0.88; 95% CI, 0.78, 1.00, *p* = 0.049) and any stroke (OR_SD_, 0.86; 95% CI, 0.76, 0.97, *p* = 0.012) (Supplementary Figure 1) as well as between higher genetically predicted vitamin B6 levels and lower risk of ischemic stroke (OR_SD_, 0.88; 95% CI, 0.81, 0.97, *p* = 0.009) and higher risk of peripheral artery disease (OR_SD_, 1.30; 95% CI, 1.09, 1.54, *p* = 0.004) (Supplementary Figure 2). Genetically predicted vitamin B12 levels were not associated with any CVD (Supplementary Figure 3 and Supplementary Table 5 and 7).

## Discussion

This MR study investigated the potential causal role of circulating tHcy and B vitamins in a broad range of CVDs and revealed suggestive associations of higher genetically predicted tHcy levels with increased risk of any stroke, subarachnoid hemorrhage, and ischemic stroke. Furthermore, higher genetically predicted levels of folate and vitamin B6 were suggestively associated with a reduced risk of any stroke and ischemic stroke, respectively. Higher genetically predicted folate levels were additionally associated with a suggestive lower risk of coronary artery disease, whereas genetically predicted vitamin B6 levels showed a suggestive positive association with risk of peripheral artery disease. There was no evidence in support of any association between genetically predicted vitamin B12 levels and the 12 studied CVDs.

The detrimental role of tHcy in stroke, especially in ischemic stroke and small vessel disease, has been established in a large body of observational studies [3, 10], MR studies [13, 14, 17] and RCTs [42]. The present study confirmed such potential benefit of homocysteine-lowing therapy with B vitamins in the primary prevention of stroke. Nevertheless, this study did not support an association between genetically predicted vitamin B12 and stroke, which is in line with results of subgroup analyses for vitamin B12 supplementation and baseline blood vitamin B12 levels in relation to risk of stroke in a meta-analysis of 14 RCTs [10]. A possible explanation may be that vitamin B12 generates little impact on tHcy levels [17]. In a meta-analysis of 12 RCTs, daily folic acid (synthetic form of folate) supplementation reduced blood homocysteine levels by 25% and vitamin B12 supplementation produced an additional 7% reduction in blood homocysteine [43].

From a mechanistic perspective, homocysteinemia may increase the risk of ischemic stroke via several pathways. Excessive homocysteine can directly impair neuronal cells and blood-brain barrier function by promoting oxidative stress, protein homocysteinylation, and Ca^2+^ dysregulation. In addition, homocysteine can induce deoxyribonucleic acid hypomethylation and worsen apoptosis, neuronal death, and blood-brain barrier dysregulation [44]. Such actions can promote damage to brain parenchyma and susceptibility to damage from ischemic stroke [44]. Furthermore, the intact vascular endothelium is integral for preventing cardiovascular sequelae such as ischemic stroke, and high levels of homocysteine can also promote endothelial dysfunction in multiple ways. This includes increased oxidant stress, decreased bioavailability of nitric oxide, increased endothelial inflammation with expression of vascular adhesion molecules and leukocyte recruitment, increased platelet activation, and promotion of thrombosis [1, 45]. Together these consequences of the dysfunctional endothelium promote all stages of ischemic stroke from early atherosclerosis development through to thrombosis and this is likely to be an important mechanism contributing to the excess ischemic stroke risk which is established in patients with high levels of homocysteine.

Studies on homocysteine in relation to hemorrhagic stroke are scarce and conflicting. A meta-analysis of two prospective studies [46] and another cohort study [47] found that blood homocysteine was not associated with the risk of hemorrhagic stroke. A positive association between blood tHcy and hemorrhagic stroke was revealed in a meta-analysis of 7 studies including 667 patients with intracerebral hemorrhage [48]. However, this positive association was not replicated in a study that found no difference in the frequency of the T allele in the SNP located in the *MTHFR* gene between intracerebral hemorrhage cases and controls [49]. In another case-control study, genetic polymorphisms of homocysteine metabolism showed an association with risk of intracranial aneurysms [50]. The present study based on more tHcy-associated SNPs and hemorrhagic stroke cases suggested that an elevated level of tHcy appeared to be a risk factor for subarachnoid hemorrhage. Nevertheless, we did not observe any clear pattern of association between genetically proxied B vitamins and subarachnoid hemorrhage. Thus, more study is warranted to confirm our findings.

Circulating levels of tHcy have been associated with risk of coronary artery disease in observational studies [51]. However, this association was not replicated in the present and previous MR studies [15, 16, 18, 19]. This discrepancy may indicate that tHcy is likely to be a risk marker instead of a causal risk factor for coronary artery disease. Notably, we observed a weak protective effect of genetically predicted folate levels on coronary artery disease risk which corroborates observational findings [52, 53], although folic acid supplementation was not found to impact coronary artery disease risk in RCTs [42]. However, folic acid improved flow-mediated endothelial vasodilator function in multiple clinical intervention trials using this as a surrogate marker for cardiovascular disease risk [54]. A protective effect of folic acid on coronary artery disease is therefore likely to be mediated by improved endothelial function [55], and importantly, many of these studies were in hyperhomocysteinemic patients specifically [56, 57]. As folate supplementation is known to reduce homocysteine levels [43], this may be one such mechanism for our finding. Other mechanisms may include the antioxidant potential of folate or its interactions with eNOS which is atheroprotective [54]. Lastly, some diagnoses of coronary artery disease may represent angina or type 2 myocardial infarctions due to anemia, and it is plausible that higher folate levels may prevent such diagnoses, although this is unlikely to explain our findings on the population level. Our finding of reduced coronary artery disease risk with higher folate levels requires further investigation and is likely to be mediated by improved endothelial function.

The present study has several strengths and limitations. The major merit is the MR design, which reinforced the causal inference by diminishing residual confounding and other biases. In addition, we investigated the associations of genetically predicted tHcy and B vitamins with CVDs using several independent data sources, which guaranteed the robustness of our findings. In addition, we combined results based on non-overlapping data sources to increase the sample size, especially for infrequent endpoints. This study also employed more SNPs, which explained more phenotypic variance, to proxy circulating levels of tHcy and B vitamins. Thus, our findings should be more statistically powered even though certain weak associations might still have been overlooked. We confined the population in the present study to individuals of European ancestry to minimize population structure bias, with the exception for the analysis for coronary artery disease, which might be challenged by bias from ethnicity, based on consortium data where European individuals comprised over 80% of participants. Nevertheless, this population confinement limited the generalizability of our findings to other populations.

The instrumental variables for folate and vitamin B12 have been validated by using pernicious anemia and mean corpuscular volume as positive controls [58]. With regard to tHcy, several of its related SNPs are located in genes (e.g., *MTHFR*, *MTR,* and *CBS*) encoding enzymes in the metabolism of homocysteine. However, whether the SNP for vitamin B6 was valid remained uncertain given that no suitable positive control was tested, and the corresponding SNP was derived from a GWAS based on a small sample size [31], albeit with replication in another GWAS [59]. Thus, the observed associations for genetically predicted vitamin B6 need verification, especially the positive association for peripheral artery disease, which is conflicting with the observational finding [53]. Another limitation is the possibility of pleiotropy. However, our sensitivity analyses generated directionally consistent results albeit with wider CIs caused by inadequate power [33], and the MR-Egger regression (not for the analyses of folate and vitamin B6 due to few SNPs) indicated no pleiotropic effect in most analyses, which suggest that pleiotropy did not bias our results. In detail, some tHcy-associated SNPs influence the genetically predisposition to other cardiovascular risk factors, such as blood pressure and high-density lipoprotein cholesterol [17]. These factors exert pan-effects on a wide range of cardiovascular disease. Thus, a few of specific associations detected in the present study were less likely to be driven by these pleiotropic effects. In addition, high blood pressure and imbalanced lipids fraction might be consequence of endothelial dysfunction as well as altered lipoprotein metabolism caused by high levels of homocysteine [1]. Whether these traits belonged to horizontal pleiotropic factors or mediators named as horizontal pleiotropic factor in MR remained unknown. Canalization (e.g., genetic buffering or developmental compensation) might bias the MR results; however, the magnitude of this issue in MR analysis has not been well-understood yet [60]. In addition, we did multiple-testing adjustment based on the Bonferroni method, which might be too stringent to inflate risk of false-negative findings (type 2 errors).

## Conclusions

This MR study provides limited evidence in support of a general benefit of lowering tHcy levels in the prevention of a broad range of CVDs in the general population. Nevertheless, our findings confirm and extend the evidence that B vitamin therapy lowering tHcy may play a role in the prevention of stroke, especially ischemic stroke and possibly subarachnoid hemorrhage.

## Supplementary Information

**Additional file 1.**

## Data Availability

All data analyzed in this study are available OSF data respiratory (https://osf.io/527zy/).
