# Supplementary material for: Homocysteine, B vitamins, and cardiovascular disease: a Mendelian randomization study
Source: BMC Med. 2021 Apr 23;19:97. doi: 10.1186/s12916-021-01977-8 (PMC8063383; doi:10.1186/s12916-021-01977-8)
Supplement: Supplementary file 1 — Additional file 1. [file 12916_2021_1977_MOESM1_ESM.docx]

**Supporting materials for**

**Homocysteine, B vitamins and Cardiovascular Disease: A Mendelian Randomization Study**

*Shuai Yuan, Paul Carter, Amy M. Mason, Stephen Burgess, Susanna C. Larsson*

**Supplementary Table 1.** Information on instrumental variables

**Supplementary Table 2.** Pleiotropic associations with used SNPs for homocysteine

**Supplementary Table 3.** Power estimation

**Supplementary Table 4.** Associations of genetically predicted circulating homocysteine with risk of cardiovascular disease in sensitivity analyses

**Supplementary Table 5.** Associations of genetically predicted circulating homocysteine and vitamin B12 with risk of cardiovascular disease in the MR-PRESSO analysis

**Supplementary Table 6.** Associations of genetically predicted circulating homocysteine with risk of cardiovascular disease in the sensitivity analysis with exclusion of 4 pleotropic SNPs

**Supplementary Table 7.** Associations of genetically predicted circulating vitamin B12 with risk of cardiovascular disease in sensitivity analyses

**Supplementary Figure 1.** Associations of genetically predicted circulating folate levels with cardiovascular disease

**Supplementary Figure 2.** Associations of genetically predicted circulating vitamin B6 levels with cardiovascular disease

**Supplementary Figure 3.** Associations of genetically predicted circulating vitamin B12 levels with cardiovascular disease

**Supplementary Table 1.** Information on instrumental variables

| **Exposure** | **SNP** | **Chr** | **Position** | **EA** | **NEA** | **EAF** | **Beta** | **SE** | **P value** | **Nearby gene** |
| --- | --- | --- | --- | --- | --- | --- | --- | --- | --- | --- |
| Vitamin B6 | rs4654748 | 1 | 21786068 | T | C | 0.50 | 1.450 | 0.280 | 8.30E-18 | *ALPL* |
| Vitamin B12 | rs2336573 | 19 | 8367709 | T | C | 0.03 | 0.320 | 0.007 | 8.40E-59 | *CD320* |
| Vitamin B12 | rs1131603 | 22 | 31018975 | C | T | 0.06 | 0.190 | 0.017 | 4.90E-49 | *TCN2* |
| Vitamin B12 | rs3742801 | 14 | 74759006 | T | C | 0.29 | 0.045 | 0.009 | 1.70E-13 | *ABCD4* |
| Vitamin B12 | rs2270655 | 4 | 146576418 | G | C | 0.94 | 0.066 | 0.018 | 2.20E-13 | *MMAA* |
| Vitamin B12 | rs12272669 | 11 | 71392610 | A | G | 0.01 | 0.510 | 0.007 | 3.00E-09 | *MMACHC* |
| Vitamin B12 | rs34324219 | 11 | 59623378 | C | A | 0.88 | 0.210 | 0.007 | 1.10E-111 | *TCN1* |
| Vitamin B12 | rs34528912 | 11 | 59631535 | T | C | 0.04 | 0.170 | 0.021 | 2.10E-15 | *TCN1* |
| Vitamin B12 | rs117456053 | 11 | 59616831 | G | A | 0.98 | 0.160 | 0.026 | 1.90E-09 | *TCN1* |
| Vitamin B12 | rs7788053 | 7 | 86773722 | A | G | 0.25 | 0.046 | 0.007 | 1.70E-10 | *FUT6* |
| Vitamin B12 | rs602662 | 19 | 49206985 | A | G | 0.60 | 0.160 | 0.007 | 2.40E-139 | *FUT2* |
| Vitamin B12 | rs1801222 | 10 | 17156151 | G | A | 0.59 | 0.110 | 0.007 | 3.30E-75 | *CUBN* |
| Vitamin B12 | rs56077122 | 10 | 17207015 | A | C | 0.34 | 0.087 | 0.009 | 4.80E-21 | *CUBN/TRDMT1* |
| Vitamin B12 | rs41281112 | 13 | 100518634 | C | T | 0.95 | 0.170 | 0.020 | 8.90E-35 | *CLYBL* |
| Vitamin B12 | rs1141321 | 6 | 49412433 | C | T | 0.63 | 0.061 | 0.007 | 3.60E-26 | *MUT* |
| Folate | rs652197 | 11 | 71849741 | C | T | 0.18 | 0.069 | 0.011 | 1.40E-12 | *FOLR3* |
| Folate | rs1801133 | 1 | 11856378 | G | A | 0.67 | 0.096 | 0.008 | 9.50E-53 | *MTHFR* |
| Homocysteine | rs1801133 | 1 | 11856378 | A | G | 0.34 | 0.158 | 0.007 | 4.30E-104 | *MTHFR* |
| Homocysteine | rs2275565 | 1 | 237048676 | G | T | 0.79 | 0.054 | 0.009 | 2.00E-10 | *MTR* |
| Homocysteine | rs1047891 | 2 | 211540507 | A | C | 0.33 | 0.086 | 0.008 | 4.60E-27 | *CPS1* |
| Homocysteine | rs9369898 | 6 | 49382193 | A | G | 0.62 | 0.045 | 0.007 | 2.20E-10 | *MUT* |
| Homocysteine | rs7130284 | 11 | 89148372 | C | T | 0.93 | 0.124 | 0.013 | 1.90E-20 | *NOX4* |
| Homocysteine | rs154657 | 16 | 89708096 | A | G | 0.47 | 0.096 | 0.007 | 1.70E-43 | *DPEP1* |
| Homocysteine | rs234709 | 21 | 44486964 | C | T | 0.55 | 0.072 | 0.007 | 3.90E-24 | *CBS* |
| Homocysteine | rs4660306 | 1 | 45978675 | T | C | 0.33 | 0.043 | 0.007 | 2.30E-09 | *MMACHC* |
| Homocysteine | rs548987 | 6 | 25869371 | C | G | 0.13 | 0.060 | 0.010 | 1.10E-08 | *SLC17A3* |
| Homocysteine | rs42648 | 7 | 89977760 | G | A | 0.60 | 0.039 | 0.007 | 2.00E-08 | *GTPB10* |
| Homocysteine | rs1801222 | 10 | 17156151 | A | G | 0.34 | 0.045 | 0.007 | 8.40E-10 | *CUBN* |
| Homocysteine | rs2251468 | 12 | 121405126 | C | A | 0.35 | 0.051 | 0.007 | 1.30E-12 | *HNF1A* |
| Homocysteine | rs838133 | 19 | 49259529 | A | G | 0.45 | 0.042 | 0.007 | 7.50E-09 | *FUT2* |
| Homocysteine | rs12780845 | 10 | 17223244 | A | G | 0.65 | 0.053 | 0.009 | 7.80E-10 | *CUBN* |

Chr, chromosome; EA, effect allele; EAF, effect allele frequency; NEA, non-effect allele; SE, standard error; SNP, single nucleotide polymorphism.

**Supplementary Table 2.** Pleiotropic associations with used SNPs for homocysteine

| **SNP** | **Nearby gene** | **Effect allele** | **Traits** | **Direction** |
| --- | --- | --- | --- | --- |
| rs2275565 | MTR | G | NA |  |
| rs1801133 | MTHFR | A | Diastolic blood pressure | + |
|  |  |  | Mean corpuscular hemoglobin | + |
| rs4660306 | MMACHC | T | NA |  |
| rs1047891 | CPS1 | A | Whole body water mass | + |
|  |  |  | Whole body and trunk fat-free mass | + |
|  |  |  | Impedance of whole body | - |
|  |  |  | Basal metabolic rate | + |
|  |  |  | Platelet count | + \| - |
|  |  |  | Creatinine in urine | + |
|  |  |  | Chronic kidney disease | + |
|  |  |  | Weight | + |
|  |  |  | Mother's heart disease | - |
|  |  |  | Mean corpuscular hemoglobin | + |
|  |  |  | Mean corpuscular volume | + |
|  |  |  | High density lipoprotein cholesterol | + \| - |
|  |  |  | Systolic blood pressure | - |
|  |  |  | Plateletcrit | - |
|  |  |  | Eosinophil percentage of white cells | + |
|  |  |  | Fibrinogen | + |
|  |  |  | Headache | + |
|  |  |  | White blood cell count | - |
|  |  |  | Hip circumference | + |
| rs548987 | SLC17A3 | C | Mean corpuscular hemoglobin | - |
|  |  |  | Red cell distribution width | + |
|  |  |  | Hemoglobin concentration | - |
|  |  |  | Primary sclerosing cholangitis | + |
|  |  |  | Mean corpuscular volume | - |
|  |  |  | Hematocrit | - |
|  |  |  | Intestinal malabsorption | + |
|  |  |  | Reticulocyte count | - |
|  |  |  | IgA deficiency | + |
|  |  |  | Schizophrenia | - |
|  |  |  | Lymphocyte count | - |
|  |  |  | Forced expiratory volume | - |
|  |  |  | Leg fat mass | + |
|  |  |  | Monocyte count | - |
|  |  |  | Self-reported gout | + |
|  |  |  | Serum urate | + |
|  |  |  | White blood cell count | - |
|  |  |  | Self-reported sarcoidosis | + |
|  |  |  | Body mass index | + |
|  |  |  | Headache | - |
| rs9369898 | MUT | A | NA |  |
| rs42648 | GTPB10 | G | Impedance of arm right | + |
| rs1801222 | CUBN | A | NA |  |
| rs12780845 | CUBN | A | NA |  |
| rs7130284 | NOX4 | C | NA |  |
| rs2251468 | HNF1A | C | Low density lipoprotein | + |
|  |  |  | Coronary artery disease | + |
|  |  |  | Plateletcrit | - |
|  |  |  | Total cholesterol | + |
|  |  |  | Gamma glutamyl transferase | - |
|  |  |  | C-reactive protein | - |
|  |  |  | Mean corpuscular hemoglobin | + |
| rs154657 | DPEP1 | A | Self-reported hypertension | - |
|  |  |  | Mean corpuscular volume | + |
|  |  |  | Hematocrit | + |
|  |  |  | log eGFR creatinine in non-diabetics | - |
| rs838133 | FUT2 | A | Sodium in urine | - |
|  |  |  | Mean platelet volume | - |
|  |  |  | Hip circumference | - |
|  |  |  | Sitting height | - |
|  |  |  | Percentage of total caloric intake from macronutrients protein | ? |
|  |  |  | Dietary macronutrient intake | - |
|  |  |  | Total cholesterol | + |
|  |  |  | Cholelithiasis | + |
| rs234709 | CBS | C | Blood and toenail selenium levels | ? |

NA, not available; SNP, single nucleotide polymorphism.

These associations were identified at the genome-wide significance level from the PhenoScanner V2, a database of human genotype-phenotype associations (http://www.phenoscanner.medschl.cam.ac.uk/).

**Supplementary Table 3.** Power estimation

| **Cardiovascular disease** | **Source** | **Total** | **Case%** | **OR_tHcy_** | **OR_B12_** | **OR_folate_** | **OR_B6_** | **~6% of variance** | | **~1% of variance** | |
| --- | --- | --- | --- | --- | --- | --- | --- | --- | --- | --- | --- |
|  |  |  |  |  |  |  |  | **OR at 80% power** | | **OR at 80% power** | |
|  |  |  |  |  |  |  |  | **≤ lower** | **≥ upper** | **≤ lower** | **≥ upper** |
| Abdominal aortic aneurysm | UKBB | 367561 | 0.005 | 1.20 | 1.12 | 1.21 | 0.53 | 0.73 | 1.27 | 0.35 | 1.66 |
| Aortic aneurysm | FinnGen | 169762 | 0.011 | 1.06 | 1.18 | 1.25 | **1.25** | 0.73 | 1.27 | 0.35 | 1.66 |
| Aortic valve stenosis | UKBB | 367561 | 0.010 | 1.14 | 0.98 | 1.12 | 1.01 | 0.81 | 1.19 | 0.54 | 1.47 |
| Atrial fibrillation | Consortium (Nielsen et al) | 1030836 | 0.059 | 0.97 | 0.99 | 1.07 | 0.93 | 0.95 | 1.05 | 0.88 | 1.12 |
| Atrial fibrillation | FinnGen | 114539 | 0.151 | 0.91 | 1.00 | 1.23 | 1.02 | 0.91 | 1.10 | 0.77 | 1.24 |
| Coronary artery disease | CARDIoGRAMplusC4D+UKBB | 547261 | 0.224 | 1.08 | 1.01 | 0.87 | 0.94 | 0.96 | 1.04 | 0.91 | 1.09 |
| Coronary artery disease | FinnGen | 176899 | 0.094 | 1.01 | 1.00 | 0.97 | 1.12 | 0.91 | 1.10 | 0.77 | 1.24 |
| Heart failure | HERMES | 977323 | 0.048 | 0.97 | 1.04 | 1.01 | 0.93 | 0.94 | 1.06 | 0.87 | 1.13 |
| Heart failure | FinnGen | 168862 | 0.057 | 0.94 | 0.97 | 1.21 | 0.92 | 0.88 | 1.12 | 0.71 | 1.30 |
| Intracerebral hemorrhage | ISGC | 6948 | 0.464 | 0.92 | - | - | - | 0.75 | 1.31 | 0.50 | 1.91 |
| Intracerebral hemorrhage | UKBB | 367561 | 0.004 | 1.03 | 0.93 | 0.90 | 1.02 | 0.75 | 1.25 | 0.27 | 1.73 |
| Intracerebral hemorrhage | FinnGen | 164757 | 0.007 | 1.26 | 1.13 | 0.95 | 1.46 | 0.66 | 1.34 | 0.18 | 1.83 |
| Ischemic stroke | MEGASTROKE | NA | NA | 1.09 | 1.01 | 0.83 | 0.90 | NA | NA | NA | NA |
| Ischemic stroke | UKBB | 367561 | 0.018 | 1.27 | 0.97 | 0.79 | 0.82 | 1.19 | 0.81 | 0.65 | 1.35 |
| Ischemic stroke | FinnGen | 172332 | 0.047 | 0.98 | 1.00 | 1.23 | 0.91 | 0.87 | 1.13 | 0.68 | 1.33 |
| Peripheral arterial disease | UKBB | 367561 | 0.012 | 1.16 | 0.98 | 0.76 | 1.15 | 0.76 | 1.24 | 0.58 | 1.43 |
| Peripheral arterial disease | FinnGen | 173166 | 0.031 | 0.98 | 0.98 | 0.83 | 1.46 | 0.84 | 1.16 | 0.61 | 1.39 |
| Stroke | MEGASTROKE | 446696 | 0.091 | 1.06 | 1.07 | 0.81 | 0.90 | 0.94 | 1.06 | 0.85 | 1.15 |
| Stroke | UKBB | 367561 | 0.033 | 1.17 | 1.00 | 0.93 | 0.88 | 0.86 | 1.14 | 0.74 | 1.26 |
| Stroke | FinnGen | 147198 | 0.096 | 1.11 | 1.04 | 0.92 | 1.10 | 0.90 | 1.10 | 0.75 | 1.25 |
| Subarachnoid hemorrhage | Consortium (Bakker et al) | 79429 | 0.094 | 1.17 | - | - | 1.01 | 0.86 | 1.15 | 0.67 | 1.35 |
| Subarachnoid hemorrhage | UKBB | 367561 | 0.004 | - | 1.13 | 1.59 | 0.90 | 0.75 | 1.25 | 0.27 | 1.73 |
| Subarachnoid hemorrhage | FinnGen | 164527 | 0.006 | 1.58 | 1.14 | 0.43 | 1.05 | 0.64 | 1.36 | 0.11 | 1.90 |
| Thoracic aortic aneurysm | UKBB | 367561 | 0.002 | 0.97 | 0.84 | 0.47 | 0.89 | 0.44 | 1.56 | 0.01 | 2.03 |
| Transient ischemic attack | UKBB | 367561 | 0.013 | 1.18 | 1.08 | 0.84 | 0.85 | 0.78 | 1.22 | 0.59 | 1.42 |
| Transient ischemic attack | FinnGen | 171015 | 0.039 | 1.13 | 1.07 | 0.94 | 1.17 | 0.85 | 1.15 | 0.65 | 1.35 |
| Venous thromboembolism | UKBB | 367561 | 0.045 | 1.00 | 1.04 | 1.01 | 0.93 | 0.91 | 1.09 | 0.78 | 1.23 |
| Venous thromboembolism | FinnGen | 176899 | 0.039 | 1.20 | 1.07 | 0.92 | 0.90 | 0.86 | 1.14 | 0.65 | 1.35 |

ORs were obtained from the random-effects invariance weighted median model.

Power was calculated using an online tool: <http://cnsgenomics.com/shiny/mRnd/>.

**Supplementary Table 4.** Associations of genetically predicted circulating homocysteine with risk of cardiovascular disease in sensitivity analyses

| **Source** | **Cardiovascular disease** | **SNPs**  **used** | ***I^2^*** | ***P (I^2^)*** | **Weighted median** | | | **MR-Egger** | | | |
| --- | --- | --- | --- | --- | --- | --- | --- | --- | --- | --- | --- |
|  |  |  |  |  | **OR** | **95% CI** | ***P*** | **OR** | **95% CI** | ***P*** | ***P_intercept_*** |
| UKBB | Abdominal aortic aneurysm | 14 | 0 | 0.635 | 1.09 | 0.75, 1.58 | 0.641 | 0.93 | 0.51, 1.71 | 0.815 | 0.357 |
| UKBB | Thoracic aortic aneurysm | 14 | 0 | 0.487 | 1.13 | 0.61, 2.10 | 0.688 | 1.40 | 0.51, 3.83 | 0.508 | 0.426 |
| FinnGen | Aortic aneurysm | 13 | 34 | 0.108 | 1.00 | 0.68, 1.47 | 0.984 | 1.08 | 0.49, 2.37 | 0.846 | 0.947 |
| UKBB | Aortic valve stenosis | 14 | 55 | 0.007 | 0.97 | 0.73, 1.29 | 0.849 | 0.75 | 0.42, 1.36 | 0.350 | 0.127 |
| Consortium (Nielsen et al) | Atrial fibrillation | 14 | 11 | 0.330 | 0.96 | 0.89, 1.04 | 0.309 | 0.91 | 0.81, 1.03 | 0.128 | 0.279 |
| FinnGen | Atrial fibrillation | 13 | 7 | 0.379 | 0.83 | 0.70, 1.00 | 0.050 | 0.73 | 0.54, 0.98 | 0.034 | 0.101 |
| CARDIoGRAMplusC4D+UKBB | Coronary artery disease | 14 | 77 | <0.001 | 1.11 | 1.02, 1.21 | 0.018 | 0.95 | 0.74, 1.22 | 0.675 | 0.266 |
| FinnGen | Coronary artery disease | 13 | 34 | 0.113 | 0.97 | 0.83, 1.15 | 0.756 | 0.83 | 0.61, 1.13 | 0.232 | 0.152 |
| HERMES | Heart failure | 14 | 65 | 0.000 | 0.97 | 0.89, 1.06 | 0.519 | 0.97 | 0.76, 1.24 | 0.805 | 0.985 |
| FinnGen | Heart failure | 13 | 27 | 0.169 | 0.92 | 0.77, 1.10 | 0.363 | 0.80 | 0.56, 1.14 | 0.222 | 0.310 |
| ISGC | Intracerebal hemorrhage | 12 | 17 | 0.281 | 0.73 | 0.34, 1.56 | 0.417 | 0.97 | 0.15, 6.15 | 0.973 | 0.957 |
| UKBB | Intracerebal hemorrhage | 14 | 0 | 0.536 | 1.08 | 0.73, 1.59 | 0.698 | 1.12 | 0.59, 2.11 | 0.727 | 0.777 |
| FinnGen | Intracerebral hemorrhage | 13 | 7 | 0.375 | 1.04 | 0.67, 1.63 | 0.852 | 0.56 | 0.27, 1.19 | 0.132 | 0.017 |
| MEGASTROKE | Ischemic stroke | 14 | 42 | 0.051 | 1.13 | 1.01, 1.26 | 0.032 | 1.14 | 0.89, 1.45 | 0.293 | 0.669 |
| UKBB | Ischemic stroke | 14 | 30 | 0.139 | 1.25 | 1.03, 1.51 | 0.023 | 1.04 | 0.73, 1.50 | 0.817 | 0.233 |
| FinnGen | Ischemic stroke | 13 | 56 | 0.006 | 0.87 | 0.73, 1.05 | 0.147 | 0.61 | 0.42, 0.89 | 0.009 | 0.005 |
| UKBB | Peripheral arterial disease | 14 | 50 | 0.017 | 1.13 | 0.90, 1.43 | 0.286 | 1.02 | 0.60, 1.73 | 0.955 | 0.577 |
| FinnGen | Peripheral arterial disease | 13 | 28 | 0.165 | 1.05 | 0.83, 1.33 | 0.694 | 0.97 | 0.61, 1.55 | 0.901 | 0.967 |
| MEGASTROKE | Stroke | 14 | 66 | 0.000 | 1.05 | 0.94, 1.17 | 0.374 | 1.17 | 0.91, 1.49 | 0.222 | 0.413 |
| UKBB | Stroke | 14 | 36 | 0.085 | 1.11 | 0.96, 1.27 | 0.155 | 0.94 | 0.72, 1.23 | 0.648 | 0.068 |
| FinnGen | Stroke | 13 | 62 | 0.002 | 1.07 | 0.91, 1.27 | 0.400 | 0.80 | 0.55, 1.16 | 0.240 | 0.052 |
| Consortium (Bakker et al) | Subarachnoid hemorrhage | 12 | 0 | 0.828 | 1.24 | 0.94, 1.63 | 0.123 | 1.19 | 0.75, 1.89 | 0.470 | 0.953 |
| FinnGen | Subarachnoid hemorrhage | 13 | 0 | 0.466 | 1.93 | 1.13, 3.30 | 0.017 | 2.51 | 1.11, 5.65 | 0.027 | 0.210 |
| UKBB | Transient ischemic attack | 14 | 53 | 0.010 | 1.16 | 0.92, 1.45 | 0.215 | 0.99 | 0.58, 1.68 | 0.964 | 0.462 |
| FinnGen | Transient ischemic attack | 13 | 33 | 0.116 | 1.05 | 0.85, 1.29 | 0.644 | 0.82 | 0.56, 1.18 | 0.283 | 0.056 |
| UKBB | Venous thromboembolism | 14 | 48 | 0.024 | 0.97 | 0.86, 1.10 | 0.621 | 0.99 | 0.74, 1.31 | 0.941 | 0.947 |
| FinnGen | Venous thromboembolism | 13 | 47 | 0.030 | 0.99 | 0.79, 1.23 | 0.914 | 1.03 | 0.65, 1.63 | 0.891 | 0.471 |

CARDIoGRAMplusC4D, Coronary ARtery DIsease Genome wide Replication and Meta-analysis plus The Coronary Artery Disease Genetics; CI, confidence interval; CVD, cardiovascular disease; HERMES; Heart Failure Molecular Epidemiology for Therapeutic Targets; ISGC, International Stroke Genetic Consortium; OR, odds ratio; SNP, single nucleotide polymorphism; UKBB, UK Biobank.

The *I^2^* statistic was used to present the heterogeneity among estimates for each SNPs in one analysis. The *p* value for the intercept in the MR-Egger regression was used present the pleiotropy (*p*<0.05).

The UK Biobank was included in Consortium (Nielsen et al), HERMES consortium, ISGC and Consortium (Bakker et al). **Supplementary Table 5.** Associations of genetically predicted circulating homocysteine and vitamin B12 with risk of cardiovascular disease in the MR-PRESSO analysis

| **Cardiovascular disease** | **Source** | **Homocysteine** | | | | | | | **Vitamin B12** | | | | | | |
| --- | --- | --- | --- | --- | --- | --- | --- | --- | --- | --- | --- | --- | --- | --- | --- |
|  |  | **Used SNPs** | **Outliers** | **p_glo** | **p_dis** | **OR** | **95% CI** | **p** | **Used SNPs** | **Outliers** | **p_glo** | **p_dis** | **OR** | **95% CI** | **p** |
| Abdominal aortic aneurysm | UKBB | 14 | 0 | 0.604 | NA | NA | NA | NA | 14 | 0 | 0.985 | NA | NA | NA | NA |
| Thoracic aortic aneurysm | UKBB | 14 | 0 | 0.431 | NA | NA | NA | NA | 14 | 0 | 0.270 | NA | NA | NA | NA |
| Aortic aneurysm | FinnGen | 13 | 0 | 0.128 | NA | NA | NA | NA | 13 | 0 | 1.000 | NA | NA | NA | NA |
| Aortic valve stenosis | UKBB | 14 | 2 | 0.378 | 0.004 | 1.29 | 0.98, 1.69 | 0.091 | 14 | 0 | 0.774 | NA | NA | NA | NA |
| Atrial fibrillation | Consortia | 14 | 0 | 0.427 | NA | NA | NA | NA | 14 | 0 | 0.396 | NA | NA | NA | NA |
| Atrial fibrillation | FinnGen | 13 | 0 | 0.473 | NA | NA | NA | NA | 13 | 0 | 0.168 | NA | NA | NA | NA |
| Coronary artery disease | Consortia | 14 | 2 | <0.001 | 0.832 | 1.07 | 0.99, 1.15 | 0.096 | 13 | 2 | 0.006 | 0.199 | 1.00 | 0.96, 1.05 | 0.929 |
| Coronary artery disease | FinnGen | 13 | 0 | 0.150 | NA | NA | NA | NA | 13 | 0 | 0.679 | NA | NA | NA | NA |
| Heart failure | Consortia | 14 | 1 | 0.002 | 0.641 | 0.95 | 0.87, 1.02 | 0.188 | 12 | 1 | 0.010 | 0.337 | 1.09 | 1.02, 1.16 | 0.028 |
| Heart failure | FinnGen | 13 | 0 | 0.253 | NA | NA | NA | NA | 13 | 0 | 0.920 | NA | NA | NA | NA |
| Intracerebral hemorrhage | Consortia | 12 | 0 | 0.272 | NA | NA | NA | NA | - | - | - | - | - | - | - |
| Intracerebral hemorrhage | UKBB | 14 | 0 | 0.602 | NA | NA | NA | NA | 14 | 0 | 0.418 | NA | NA | NA | NA |
| Intracerebral hemorrhage | FinnGen | 13 | 0 | 0.431 | NA | NA | NA | NA | 13 | 0 | 0.932 | NA | NA | NA | NA |
| Ischemic stroke | Consortia | 14 | 0 | 0.145 | NA | NA | NA | NA | 13 | 1 | 0.026 | 0.833 | 1.02 | 0.95, 1.11 | 0.565 |
| Ischemic stroke | UKBB | 14 | 0 | 0.366 | NA | NA | NA | NA | 14 | 1 | 0.066 | 0.083 | 1.01 | 0.92, 1.10 | 0.908 |
| Ischemic stroke | FinnGen | 13 | 1 | 0.760 | 0.015 | 0.95 | 0.78, 1.14 | 0.560 | 13 | 0 | 0.414 | NA | NA | NA | NA |
| Peripheral arterial disease | UKBB | 14 | 0 | 0.050 | NA | NA | NA | NA | 14 | 0 | 0.407 | NA | NA | NA | NA |
| Peripheral arterial disease | FinnGen | 13 | 0 | 0.182 | NA | NA | NA | NA | 13 | 0 | 0.610 | NA | NA | NA | NA |
| Stroke | Consortia | 14 | 3 | 0.004 | <0.001 | 0.98 | 0.9, 1.06 | 0.604 | 11 | 2 | 0.044 | 0.562 | 1.09 | 1.04, 1.15 | 0.010 |
| Stroke | UKBB | 14 | 0 | 0.184 | NA | NA | NA | NA | 14 | 1 | 0.606 | 0.042 | 0.95 | 0.89, 1.01 | 0.151 |
| Stroke | FinnGen | 13 | 0 | 0.006 | NA | NA | NA | NA | 13 | 0 | 0.759 | NA | NA | NA | NA |
| Subarachnoid hemorrhage | Consortia | 12 | 0 | 0.868 | NA | NA | NA | NA | - | - | - | - | - | - | - |
| Subarachnoid hemorrhage | UKBB | - | - | - | - | - | - | - | 14 | 0 | 0.841 | NA | NA | NA | NA |
| Subarachnoid hemorrhage | FinnGen | 13 | 0 | 0.527 | NA | NA | NA | NA | 13 | 0 | 0.999 | NA | NA | NA | NA |
| Transient ischemic attack | UKBB | 14 | 2 | 0.646 | 0.032 | 1.22 | 1.04, 1.43 | 0.035 | 14 | 0 | 0.343 | NA | NA | NA | NA |
| Transient ischemic attack | FinnGen | 13 | 0 | 0.177 | NA | NA | NA | NA | 13 | 0 | 0.888 | NA | NA | NA | NA |
| Venous thromboembolism | UKBB | 14 | 2 | 0.693 | 0.038 | 0.93 | 0.87, 1 | 0.067 | 14 | 1 | 0.002 | 0.064 | 1.00 | 0.94, 1.06 | 0.943 |
| Venous thromboembolism | FinnGen | 13 | 1 | 0.167 | 0.043 | 1.11 | 0.92, 1.33 | 0.287 | 13 | 0 | 0.951 | NA | NA | NA | NA |

CI, confidence interval; NA, not available; OR, odds ratio; p_glo, p value for global test; p_dis, p value for distortion test; SNPs, singe nucleotide polymorphisms; UKBB, UK Biobank.

**Supplementary Table 6.** Associations of genetically predicted circulating homocysteine with risk of cardiovascular disease in the sensitivity analysis with exclusion of 4 pleotropic SNPs

| **Cardiovascular disease** | **Source** | **OR** | **95% CI** | **p** |
| --- | --- | --- | --- | --- |
| Aortic aneurysm | UKBB | 1.19 | 0.92, 1.54 | 0.189 |
| Aortic aneurysm | FinnGen | 0.89 | 0.65, 1.21 | 0.453 |
| Aortic aneurysm | Meta-analysis | 1.06 | 0.87, 1.29 | 0.591 |
| Aortic valve stenosis | UKBB | 1.03 | 0.76, 1.39 | 0.846 |
| Atrial fibrillation | Consortia | 0.97 | 0.91, 1.03 | 0.268 |
| Atrial fibrillation | FinnGen | 0.89 | 0.76, 1.03 | 0.121 |
| Atrial fibrillation | Meta-analysis | 0.96 | 0.90, 1.01 | 0.109 |
| Coronary artery disease | Consortia | 1.05 | 0.98, 1.14 | 0.160 |
| Coronary artery disease | FinnGen | 0.99 | 0.86, 1.13 | 0.847 |
| Coronary artery disease | Meta-analysis | 1.04 | 0.97, 1.11 | 0.256 |
| Heart failure | Consortia | 0.95 | 0.87, 1.03 | 0.180 |
| Heart failure | FinnGen | 0.91 | 0.75, 1.12 | 0.379 |
| Heart failure | Meta-analysis | 0.94 | 0.87, 1.01 | 0.116 |
| Stroke | Consortia | 1.08 | 0.94, 1.24 | 0.266 |
| Stroke | UKBB | 1.20 | 1.05, 1.37 | 0.006 |
| Stroke | FinnGen | 1.12 | 0.92, 1.36 | 0.263 |
| Stroke | Meta-analysis | 1.14 | 1.04, 1.24 | 0.003 |
| Intracerebral hemorrhage | Consortia | 1.06 | 0.57, 1.96 | 0.856 |
| Intracerebral hemorrhage | UKBB | 1.17 | 0.86, 1.61 | 0.318 |
| Intracerebral hemorrhage | FinnGen | 1.30 | 0.81, 2.07 | 0.272 |
| Intracerebral hemorrhage | Meta-analysis | 1.19 | 0.93, 1.51 | 0.162 |
| Subarachnoid hemorrhage | Consortia | 1.16 | 0.92, 1.45 | 0.206 |
| Subarachnoid hemorrhage | FinnGen | 1.68 | 1.10, 2.55 | 0.015 |
| Subarachnoid hemorrhage | Meta-analysis | 1.26 | 1.03, 1.53 | 0.024 |
| Ischemic stroke | Consortia | 1.11 | 0.97, 1.26 | 0.116 |
| Ischemic stroke | UKBB | 1.32 | 1.14, 1.54 | <0.001 |
| Ischemic stroke | FinnGen | 0.95 | 0.76, 1.19 | 0.663 |
| Ischemic stroke | Meta-analysis | 1.15 | 1.04, 1.27 | 0.002 |
| Transient ischemic attack | UKBB | 1.12 | 0.90, 1.38 | 0.306 |
| Transient ischemic attack | FinnGen | 1.14 | 0.95, 1.36 | 0.158 |
| Transient ischemic attack | Meta-analysis | 1.13 | 0.99, 1.29 | 0.082 |
| Peripheral arterial disease | UKBB | 1.13 | 0.93, 1.37 | 0.214 |
| Peripheral arterial disease | FinnGen | 1.03 | 0.84, 1.25 | 0.798 |
| Peripheral arterial disease | Meta-analysis | 1.08 | 0.94, 1.23 | 0.286 |
| Venous thromboembolism | UKBB | 0.96 | 0.87, 1.05 | 0.355 |
| Venous thromboembolism | FinnGen | 1.10 | 0.93, 1.30 | 0.280 |
| Venous thromboembolism | Meta-analysis | 0.99 | 0.91, 1.08 | 0.798 |

CI, confidence interval; OR, odds ratio; UKBB, UK Biobank.

Estimates were derived from the random-effect inverse-variance weighted model.

**Supplementary Table 7.** Associations of genetically predicted circulating vitamin B12 with risk of cardiovascular disease in sensitivity analyses

| **Source** | **Cardiovascular disease** | **SNPs**  **used** | ***I^2^*** | ***P (I^2^)*** | **Weighted median** | | | **MR-Egger** | | | |
| --- | --- | --- | --- | --- | --- | --- | --- | --- | --- | --- | --- |
|  |  |  |  |  | **OR** | **95% CI** | ***P*** | **OR** | **95% CI** | ***P*** | ***P_intercept_*** |
| UKBB | Abdominal aortic aneurysm | 14 | 0 | 0.986 | 1.04 | 0.84, 1.28 | 0.741 | 1.04 | 0.79, 1.36 | 0.800 | 0.498 |
| UKBB | Thoracic aortic aneurysm | 14 | 9 | 0.357 | 0.63 | 0.42, 0.93 | 0.022 | 0.80 | 0.49, 1.30 | 0.365 | 0.799 |
| FinnGen | Aortic aneurysm | 13 | 0 | 0.910 | 1.15 | 0.94, 1.39 | 0.176 | 1.14 | 0.89, 1.46 | 0.311 | 0.733 |
| UKBB | Aortic valve stenosis | 14 | 0 | 0.769 | 1.01 | 0.87, 1.17 | 0.865 | 1.03 | 0.86, 1.25 | 0.720 | 0.441 |
| Consortium (Nielsen et al) | Atrial fibrillation | 14 | 8 | 0.365 | 1.01 | 0.96, 1.06 | 0.771 | 0.95 | 0.88, 1.03 | 0.192 | 0.226 |
| FinnGen | Atrial fibrillation | 13 | 38 | 0.078 | 0.99 | 0.90, 1.09 | 0.833 | 0.99 | 0.85, 1.17 | 0.945 | 0.983 |
| CARDIoGRAMplusC4D+UKBB | Coronary artery disease | 13 | 61 | 0.002 | 1.01 | 0.97, 1.06 | 0.501 | 1.00 | 0.92, 1.09 | 0.982 | 0.840 |
| FinnGen | Coronary artery disease | 13 | 0 | 0.608 | 0.98 | 0.90, 1.07 | 0.709 | 0.99 | 0.89, 1.10 | 0.851 | 0.821 |
| HERMES | Heart failure | 12 | 60 | 0.004 | 1.07 | 1.00, 1.15 | 0.047 | 0.93 | 0.79, 1.09 | 0.380 | 0.113 |
| FinnGen | Heart failure | 13 | 0 | 0.852 | 0.92 | 0.83, 1.01 | 0.080 | 0.88 | 0.78, 1.00 | 0.047 | 0.054 |
| UKBB | Intracerebal hemorrhage | 14 | 10 | 0.346 | 0.93 | 0.74, 1.18 | 0.549 | 1.01 | 0.74, 1.37 | 0.968 | 0.496 |
| FinnGen | Intracerebral hemorrhage | 13 | 3 | 0.419 | 1.22 | 0.96, 1.55 | 0.103 | 1.50 | 1.10, 2.03 | 0.010 | 0.024 |
| MEGASTROKE | Ischemic stroke | 13 | 52 | 0.015 | 1.01 | 0.93, 1.11 | 0.789 | 1.09 | 0.92, 1.28 | 0.329 | 0.311 |
| UKBB | Ischemic stroke | 14 | 34 | 0.106 | 1.00 | 0.89, 1.12 | 0.980 | 1.02 | 0.86, 1.21 | 0.833 | 0.473 |
| FinnGen | Ischemic stroke | 13 | 15 | 0.288 | 0.99 | 0.89, 1.09 | 0.830 | 0.99 | 0.86, 1.14 | 0.905 | 0.912 |
| UKBB | Peripheral arterial disease | 14 | 4 | 0.405 | 0.92 | 0.81, 1.05 | 0.233 | 0.91 | 0.77, 1.08 | 0.288 | 0.262 |
| FinnGen | Peripheral arterial disease | 13 | 0 | 0.525 | 0.92 | 0.82, 1.04 | 0.184 | 0.86 | 0.74, 1.01 | 0.063 | 0.041 |
| MEGASTROKE | Stroke | 11 | 64 | 0.002 | 1.09 | 1.01, 1.17 | 0.027 | 1.14 | 0.95, 1.37 | 0.161 | 0.408 |
| UKBB | Stroke | 14 | 44 | 0.040 | 1.01 | 0.93, 1.10 | 0.852 | 1.05 | 0.91, 1.20 | 0.509 | 0.379 |
| FinnGen | Stroke | 13 | 12 | 0.326 | 1.02 | 0.94, 1.11 | 0.670 | 1.07 | 0.95, 1.20 | 0.276 | 0.573 |
| UKBB | Subarachnoid hemorrhage | 14 | 0 | 0.813 | 1.07 | 0.84, 1.37 | 0.585 | 1.10 | 0.81, 1.50 | 0.537 | 0.842 |
| FinnGen | Subarachnoid hemorrhage | 13 | 0 | 0.977 | 1.17 | 0.91, 1.52 | 0.226 | 1.29 | 0.92, 1.80 | 0.140 | 0.353 |
| UKBB | Transient ischemic attack | 14 | 21 | 0.226 | 1.05 | 0.92, 1.19 | 0.487 | 1.10 | 0.91, 1.33 | 0.312 | 0.755 |
| FinnGen | Transient ischemic attack | 13 | 28 | 0.161 | 1.08 | 0.96, 1.20 | 0.194 | 1.11 | 0.94, 1.31 | 0.218 | 0.591 |
| UKBB | Venous thromboembolism | 14 | 41 | 0.056 | 1.00 | 0.93, 1.08 | 0.952 | 1.00 | 0.89, 1.12 | 0.992 | 0.438 |
| FinnGen | Venous thromboembolism | 13 | 8 | 0.368 | 1.07 | 0.96, 1.19 | 0.219 | 1.04 | 0.9, 1.20 | 0.619 | 0.547 |

CARDIoGRAMplusC4D, Coronary ARtery DIsease Genome wide Replication and Meta-analysis plus The Coronary Artery Disease Genetics; CI, confidence interval; CVD, cardiovascular disease; HERMES; Heart Failure Molecular Epidemiology for Therapeutic Targets; OR, odds ratio; SNP, single nucleotide polymorphism; UKBB, UK Biobank.

The *I^2^* statistic was used to present the heterogeneity among estimates for each SNPs in one analysis. The *p* value for the intercept in the MR-Egger regression was used present the pleiotropy (*p*<0.05).

The UK Biobank was included in Consortium (Nielsen et al) and HERMES consortium.

**Supplementary Figure 1.** Associations of genetically predicted serum folate levels with cardiovascular disease

**
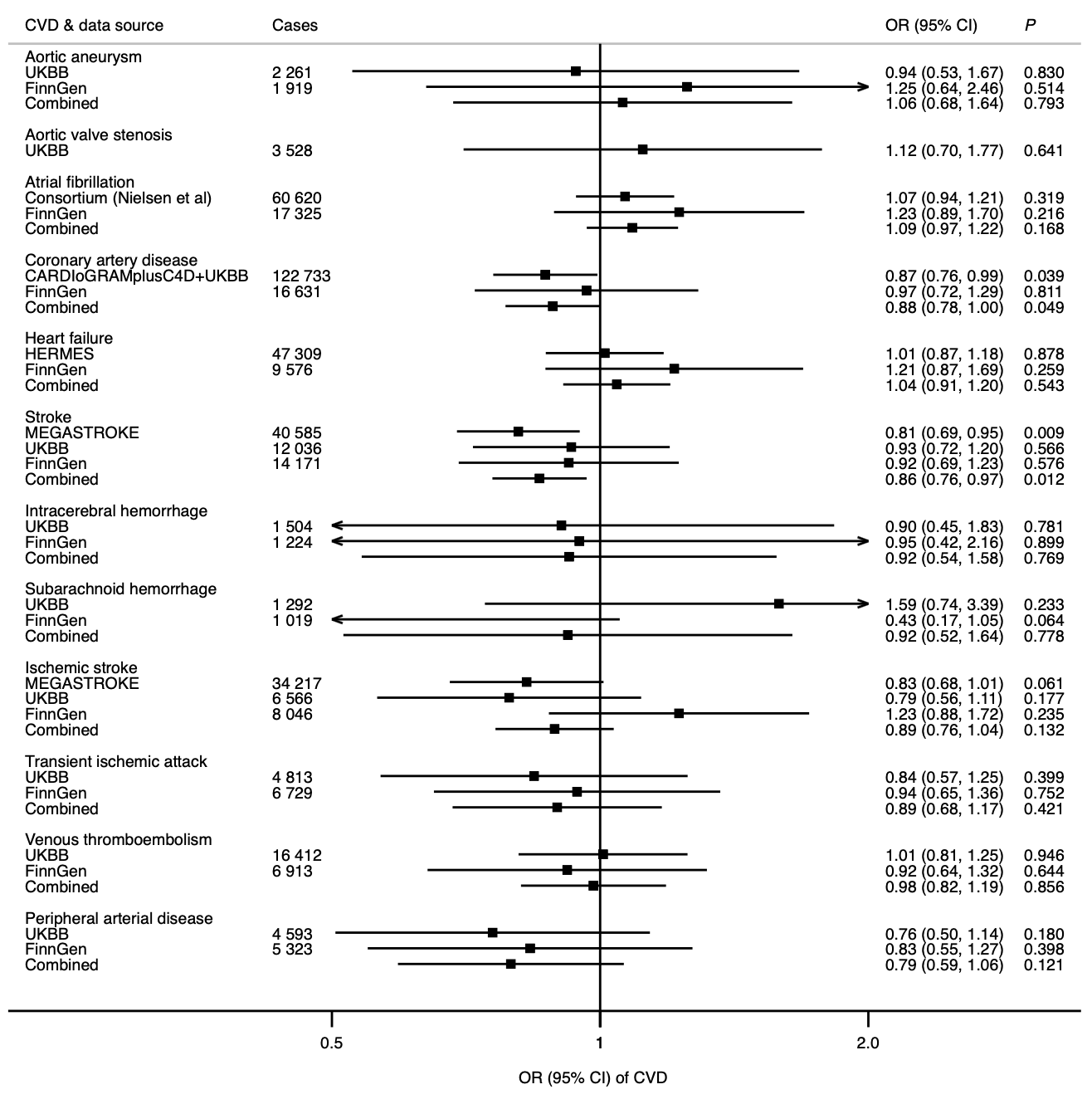
**

CARDIoGRAMplusC4D, Coronary ARtery DIsease Genome wide Replication and Meta-analysis plus The Coronary Artery Disease Genetics; CI, confidence interval; CVD, cardiovascular disease; HERMES; Heart Failure Molecular Epidemiology for Therapeutic Targets; ISGC, International Stroke Genetic Consortium; OR, odds ratio; UKBB, UK Biobank. The UK Biobank was included in Consortium (Nielsen et al), HERMES consortium, ISGC and Consortium (Bakker et al).

**Supplementary Figure 2.** Associations of genetically predicted serum vitamin B6 levels with cardiovascular disease

**
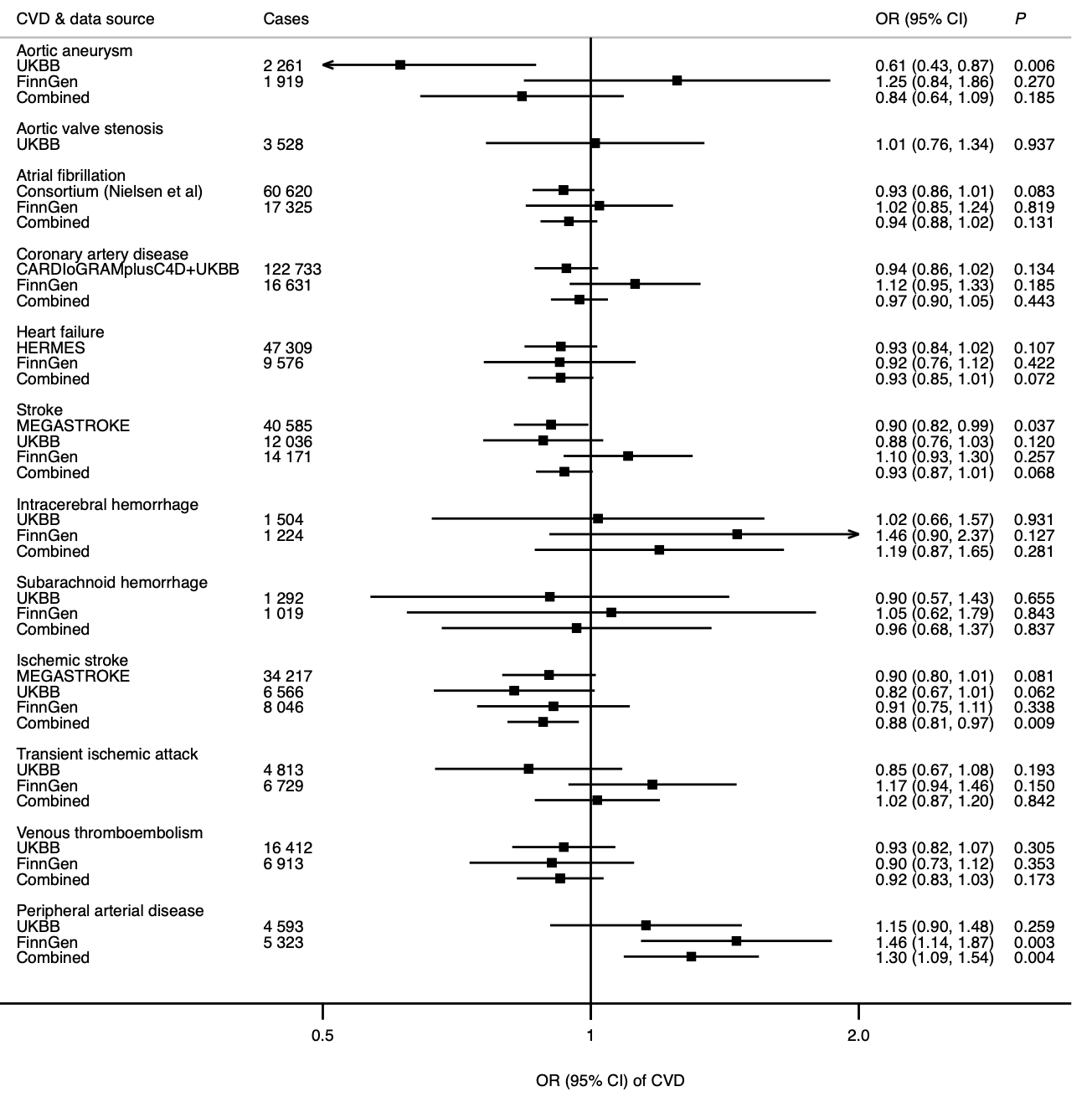
**

CARDIoGRAMplusC4D, Coronary ARtery DIsease Genome wide Replication and Meta-analysis plus The Coronary Artery Disease Genetics; CI, confidence interval; CVD, cardiovascular disease; HERMES; Heart Failure Molecular Epidemiology for Therapeutic Targets; ISGC, International Stroke Genetic Consortium; OR, odds ratio; UKBB, UK Biobank. The UK Biobank was included in Consortium (Nielsen et al), HERMES consortium, ISGC and Consortium (Bakker et al).

**Supplementary Figure 3.** Associations of genetically predicted serum vitamin B12 levels with cardiovascular disease

**
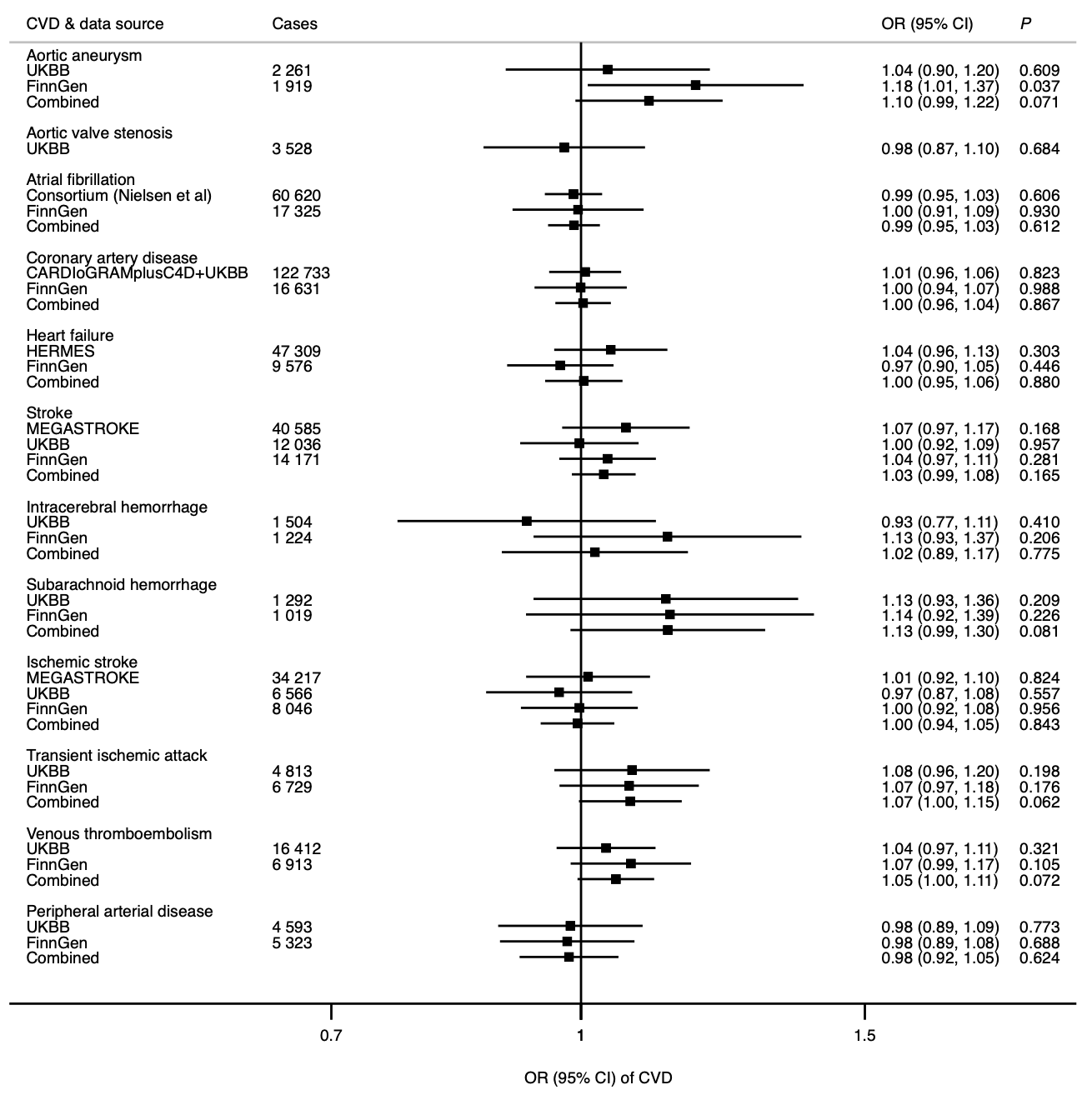
**

CARDIoGRAMplusC4D, Coronary ARtery DIsease Genome wide Replication and Meta-analysis plus The Coronary Artery Disease Genetics; CI, confidence interval; CVD, cardiovascular disease; HERMES; Heart Failure Molecular Epidemiology for Therapeutic Targets; ISGC, International Stroke Genetic Consortium; OR, odds ratio; UKBB, UK Biobank. The UK Biobank was included in Consortium (Nielsen et al), HERMES consortium, ISGC and Consortium (Bakker et al).
